# Changes of gut microbiota under different nutritional methods in elderly patients with severe COVID-19 and their relationship with prognosis

**DOI:** 10.3389/fimmu.2023.1260112

**Published:** 2023-09-13

**Authors:** Jiawei Zhang, Jiaxin Deng, Juan Li, Yuping Su, Jiancong Hu, Dezheng Lin, Mingli Su, Yongcheng Chen, Sen Liao, Xuhao Bai, Miwei Lv, Tian Xu, Qinghua Zhong, Xuefeng Guo

**Affiliations:** ^1^ Department of General Surgery (Endoscopic Surgery), The Sixth Affiliated Hospital, Sun Yat-sen University, Guangzhou, China; ^2^ Guangdong Provincial Key Laboratory of Colorectal and Pelvic Floor Diseases, The Sixth Affiliated Hospital, Sun Yat-sen University, Guangzhou, China; ^3^ Biomedical Innovation Center, The Sixth Affiliated Hospital, Sun Yat-sen University, Guangzhou, China; ^4^ Key Laboratory of Human Microbiome and Chronic Diseases (Sun Yat-sen University), Ministry of Education, Guangzhou, China; ^5^ The Medical College of Xizang Minzu University, Xianyang, Shaanxi, China

**Keywords:** gut microbiota, enteral nutrition, parenteral nutrition, COVID-19, old age

## Abstract

**Background:**

The clinical progression of individuals afflicted with severe acute respiratory syndrome coronavirus 2 (SARS-CoV-2) infection exhibits significant heterogeneity, particularly affecting the elderly population to a greater extent. Consequently, the association between nutrition and microbiota has garnered considerable interest. Hence, the objective of this study was to gather clinical data pertaining to the influence of diverse nutritional support interventions on the prognosis of geriatric patients with COVID-19, while additionally examining the fecal microbiota of these individuals to assess the repercussions of microecological alterations on their prognostic outcomes.

**Results:**

A total of 71 elderly patients diagnosed with severe COVID-19 were included in this study. These patients were subsequently divided into two groups, namely the enteral nutrition (EN) group and the parenteral nutrition (PN) group, based on the type of nutritional support therapy they received after admission. The occurrence of complications was observed in 10.4% of patients in the EN group, whereas it was significantly higher at 69.6% in the PN group (P<0.001). Furthermore, the 60-day mortality rate was 2.1% (1/48) in the EN group, while it was notably higher at 30.4% (7/23) in the PN group (P=0.001). To identify the independent predictors of 60-day mortality, stepwise logistic regression analysis was employed. Among different bacterial groups, *Enterococcus_faecium* (18.19%) and *Pseudomonas_aeruginosa* (1.91%) had higher average relative abundance in the PN group (P<0.05). However, the relative abundance of *Ruminococcus* was higher in the EN group. Further Spearman correlation analysis showed that *Enterococcus_faecium* was positively correlated with poor clinical prognosis, while *Ruminococcus* was negatively correlated with poor clinical prognosis.

**Conclusions:**

This study shows that the changes in the composition of intestinal flora in elderly COVID-19 patients receiving different nutritional support strategies may be related to different clinical outcomes. The abundance of *Enterococcus_faecium* in elderly COVID-19 patients receiving PN is significantly increased and is closely related to poor clinical outcomes. It highlights the potential of microbiome-centric interventions to mitigate and manage COVID-19 in older adults with different nutritional support options.

## Introduction

Corona Virus Disease 2019 (COVID-19), caused by the severe acute respiratory syndrome coronavirus 2 (SARS-CoV-2), is primarily characterized as a respiratory infection with respiratory symptoms being predominant in affected individuals. Nevertheless, it has been observed that gastrointestinal symptoms can also manifest in patients with SARS-CoV-2 infection, indicating the involvement of the gastrointestinal tract in the pathogenesis of COVID-19 (1, 2). Nutritional status is an important factor that directly affects the outcome of elderly COVID-19 patients and is closely related to their survival. For elderly COVID-19 patients, the implementation of PN is accompanied by huge risks and challenges, which may lead to more nutrition-related complications. Studies have increasingly demonstrated that SARS-CoV-2 infects and replicates within human small intestinal cells, specifically targeting the intestinal mucosa (3). The alteration of angiotensin-converting enzyme 2 (ACE2) receptor expression facilitates the entry of SARS-CoV-2 into host cells, thereby increasing expression in the gastrointestinal tract (4, 5). The presence of SARS-CoV-2 RNA in human feces suggests involvement of the gastrointestinal tract in viral infection (6). The clinical course of SARS-CoV-2 infection varies greatly, encompassing asymptomatic cases as well as severe respiratory disease accompanied by systemic complications (7). The elderly population is more severely impacted by COVID-19 due to age-related changes in metabolism and immune function. This demographic typically experiences high levels of inflammation, stress, catabolism, and increased energy and protein needs in their gastrointestinal tract (8). The presence of ACE2, which is highly expressed in the gastrointestinal tract, may contribute to symptoms like diarrhea, nausea, and vomiting, further exacerbating malnutrition. Additionally, the inflammatory response associated with the disease significantly affects the patients’ appetite (3).

In recent times, considerable focus has been directed towards investigating the correlation between nutrition and the microbiome. However, further empirical evidence is required to comprehensively comprehend the relative significance of various nutritional interventions in maintaining microbiome homeostasis during disease. Moreover, it is imperative to explore potential interventions that aid in restoring this homeostasis during the advanced stages of the disease, such as alternative nutritional support modalities or the utilization of probiotics and fecal microbiota transplantation. Currently, clinical practice employs nutritional support techniques encompassing EN and PN (9). EN offers several benefits in terms of preserving the integrity of the intestinal barrier, upholding intestinal immune function, preventing intestinal bacterial translocation, and mitigating gut-derived infections (10). Nevertheless, elderly patients with severe COVID-19 infection frequently experience gastrointestinal intolerance and other symptoms (11). Consequently, for this vulnerable population with a high risk of malnutrition, the implementation of nutritional support therapy emerges as a crucial determinant in the duration of their recovery (12). Moreover, it is noteworthy that patients classified as having a high nutritional risk exhibit substantially elevated mortality rates compared to those with a low nutritional risk (13). The urgency of nutrition for elderly patients afflicted with COVID-19 is paramount. Furthermore, the manifestation of gastrointestinal tract intolerance symptoms and signs can significantly impede the restoration of patients’ nutritional status. Consequently, devising diverse nutritional support therapies to effectively attain the nutritional objectives of elderly patients suffering from severe SARS-CoV-2 infection has emerged as a formidable challenge. In the context of severe SARS-CoV-2 infection in elderly patients, the binding of SARS-CoV-2 to the ACE2 receptor results in the disruption of the indigenous gut microbiota, impairment of the intestinal microbiome, and facilitation of disease progression (14). Notably, COVID-19 infected patients exhibit substantial alterations in their fecal microbiota, characterized by an increased presence of opportunistic pathogens and a reduction in beneficial commensal bacteria (15). These modifications in the gut microbiome, encompassing diminished bacterial diversity and shifts in microbial composition, have been observed to correlate with the severity of COVID-19 infection in affected patients (16). In the field of COVID-19 infection, a series of studies have been conducted on the relationship between gut microbiota and changes in gut microbiota during hospitalization, post-acute COVID-19 syndrome, and comparison of COVID-19 patients with different severity of COVID-19. However, there are few studies on gut microbiota and nutrient metabolism (15, 17, 18). Previous research has demonstrated notable alterations in gut microbiota during enteral or parenteral nutrition. Inadequate parenteral nutrition and diminished nutritional support for the intestinal mucosa can result in lymphoid tissue atrophy, compromised immune system functionality, and exacerbated bacterial translocation, along with other pathological manifestations (19).

In this study, stool samples were collected to compare clinical variables and gut bacterial composition among COVID-19 patients treated with different nutritional support. The clinical variables and gut bacterial composition of patients with COVID-19 infection were compared between different nutritional support treatment groups. The use of different nutritional support methods should be based on a comprehensive clinical evaluation, which can affect the gut microbiota, resulting in different prognoses. Hence, the objective of this study is to gather clinical data concerning the impact of diverse nutritional support therapies on the prognosis of elderly individuals afflicted with COVID-19. Additionally, it aims to conduct an in-depth analysis of the fecal microbiota of these patients to assess the influence of microecological alterations on the prognosis of elderly patients suffering from severe COVID-19.

## Materials and methods

### Ethical statement

Informed consent of all participants was obtained for this study. The collection of stool samples and data analysis were approved by the institutional ethics board of the Sixth Affiliated Hospital, Sun Yat-sen University (NO.2023ZSLYEC-231). All interventions performed on patients were based on clinical judgment, according to the needs of the patients and not for the purposes of this study. Data were analyzed anonymously. We confirm that all methods are implemented in accordance with relevant guidelines and regulations. The study was also conducted in accordance with the Declaration of Helsinki (revised in Fortaleza, Brazil, October 2013).

### Study design and population

**Figure 1:**
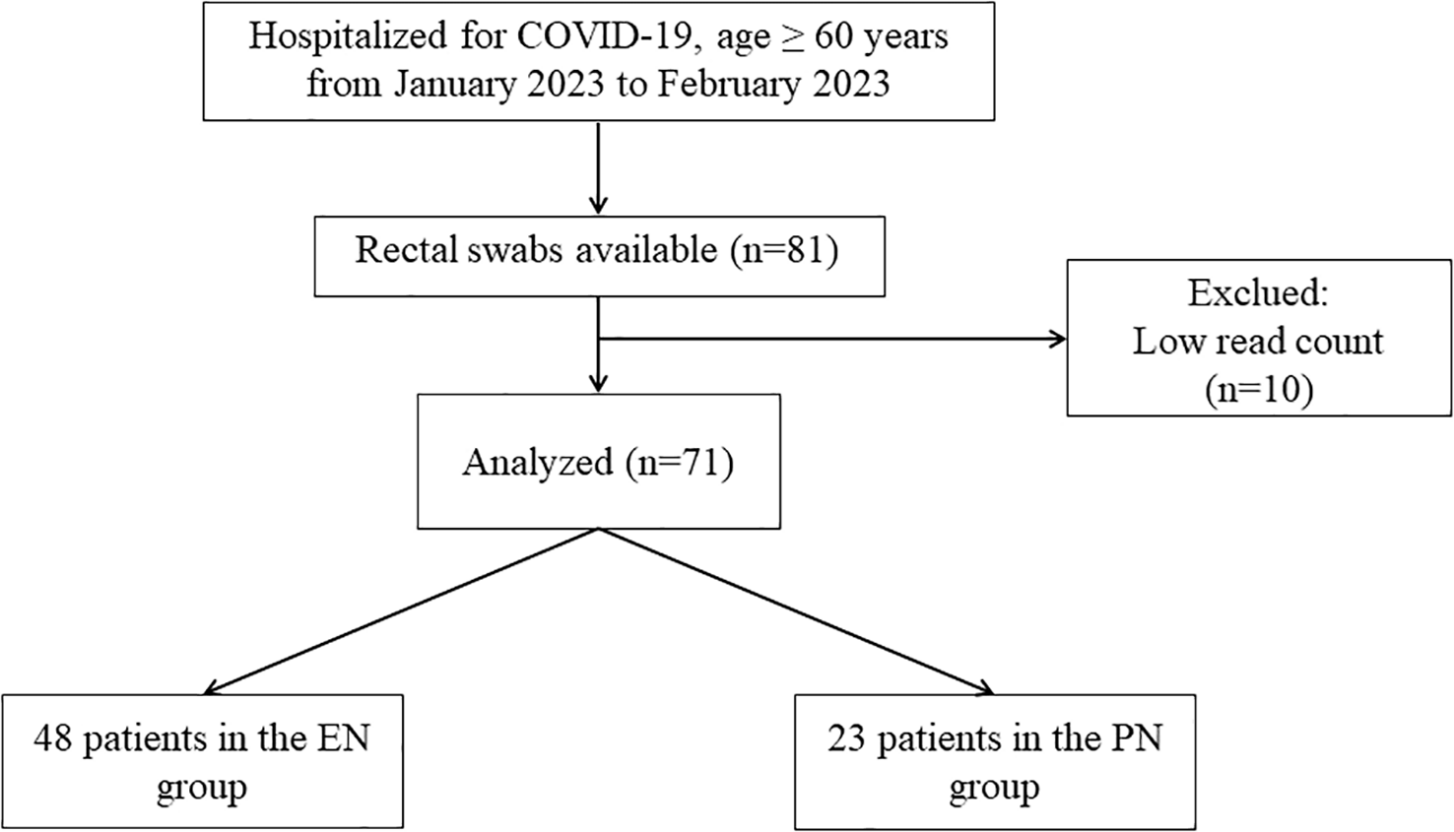
The flowchart of patient allocation.

In January and February 2023, COVID-19 patients were categorized into two groups, namely the EN group and the PN group, based on their clinical treatment requirements. Throughout their hospitalization, all participants received personalized nutritional support treatment as per the standard of care. Prior investigations have indicated a substantial alteration in the intestinal flora of patients after a week of nutritional support therapy (20, 21). Consequently, in our study, all patients were administered enteral nutrition or parenteral nutrition support for a duration of one week, following which stool samples were obtained from COVID-19 patients. Eligibility criteria for patients included being 60 years of age or older, according to the Chinese definition of the elderly individuals (22), and tested positive for SARS-CoV-2 by nasopharyngeal swabs using quantitative RT-PCR according to the recommendations of the National reference laboratory and the National Health Bureau. This study employed a prospective non-interventional design to investigate the patient inclusion process, as depicted in **Figure 1**. Within 48 hours of admission, each group received distinct forms of nutritional support. The EN group had access to oral nutrition powder, nasogastric tube, or nasojejunal access, while the PN group received parenteral nutrition *via* a central venous catheter (subclavian or jugular vein). The decision was made following a clinical evaluation of bowel function, with no rigid criteria imposed, thereby allowing the individual clinician’s discretion. Consequently, additional factors were taken into account, including the patients’ underlying condition and the anticipated rate of postoperative or intervention-related recuperation. The recommended daily intake of enteral nutrition was determined as 20-25 kcal/kg, while protein requirements were estimated at 1.5 g/kg (23, 24). The caloric intake of parenteral nutrition was ascertained to be 20-25 kcal/kg per day, while the calorie/nitrogen ratio was computed to be 120-150:1 (23). Glucose accounts for 50% to 70% of the total energy requirements, with lipid utilization contingent upon serum triglyceride levels. Furthermore, the intravenous solution was supplemented with sufficient quantities of vitamins, electrolytes, trace elements, and insulin. The medical records contained comprehensive information regarding COVID-19 patients, encompassing routine demographic data, past medical history, epidemiological exposure history, pre-existing comorbidities (such as diabetes, hypertension, chronic respiratory diseases, immunosuppression, hematological oncological disease, and previous chronic therapy), symptoms, signs, laboratory results, chest x-rays, administered antiviral therapy, corticosteroids, rescue invasive treatment, intensive care units (ICU) care, supportive measures, and in-hospital complications. Furthermore, clinical outcomes such as the duration of mechanical ventilation, ICU length of stay, and 60-day mortality were also documented.

### Sample collection and processing

Stool samples were collected from elderly COVID-19 patients as soon as possible after admission and immediately refrigerated and thawed within 24 hours and frozen at -80 °C until nucleic acid extraction. Stool samples, whether transported short or long distances, must be kept in containers filled with liquid nitrogen and supervised by humans. All samples were collected during standard care rounds using all necessary precautions. The Clinical records and laboratory information system (LIS) were used to retrieve patient data, including laboratory test results and clinical presentation.

### Stool DNA extraction and sequencing

Stool DNA was tested for concentration, integrity and purity before library construction, and the qualified samples enter the process of library construction. The gut microbiota composition was determined by 16S rRNA gene sequencing in fecal samples collected from the participants. Firstly, the sample was interrupted, and 1μg metagenomic DNA was taken, which was interrupted with a Covaris (Covaris S2 System, Massachusetts, USA) ultrasonic fragmenter. The interrupted sample magnetic beads were subjected to fragment selection so that the sample bands were concentrated around 200 to 400 bp. Then the end is repaired, “A” is added, and the connector is connected. The reaction system was prepared, the reaction was suitable for A certain period of time, the double stranded cDNA end was repaired, and the A base was added to the 3’end, the linker was prepared, the reaction system was suitable for a certain period of time, the linker was connected with the DNA. The PCR reaction system was prepared, and the reaction program was set to amplify the ligation product. The amplified products were purified and recovered by magnetic beads. After denaturation of the PCR product to a single strand, the cyclized reaction system was prepared, and the reaction was thoroughly mixed at the appropriate temperature for a certain time to obtain the single stranded circular product. After digestion of the uncyclized linear DNA molecules, the final library was obtained. A single-stranded circular DNA molecule replicates through a rolling circle to form a DNA nanosphere (DNB) containing multiple copies. The obtained DNBs were added to the mesh holes on the chip by high-density DNA nanochip technology, and sequenced by combined probe anchor polymerization (cPAS). The metagenome was sequenced using the MGISEQ 2000 platform PE150. SOAPnuke (v1.5.0) (25) software was used to filter the raw data for quality control, and Bowtie2 (2.2.5) (26) was used to align the host sequence and remove the sequence in the ratio to generate Clean Data. MEGAHIT (v1.2.9) (27) was used for *de novo* assembly of Clean Data that passed quality control. Assembled sequences less than 200 bp in length were filtered out. Species annotation was performed using Kraken2’s default parameters, while species-level abundance was estimated for metagenomic samples using a Bayesian algorithm and Kraken classification results *via* Bracken. UHGG database of human gastrointestinal genome was used for database selection (28). The sequence number of species contained in the sample was calculated by comparison between Kraken2 and self-built database (screened UHGG database), and then Bracken2 was used to estimate the actual abundance of species in the sample and complete the species annotation. The R package was used to calculate the Alpha diversity of species, including chao1 index, shannon index and simpson index. It also measures the difference between samples or groups by calculating Bray-Curtis distance and Jsen-Shannon divergence, called Beta diversity, to reflect whether there are significant microbial community differences between sample groups. Besides, linear discriminant analysis effect size (LEfSe) method was used to identify differentially rich taxa, and the LDA threshold was set to be >4 (29). Methods Nonparametric test and linear discriminant analysis were combined to find biomarkers of each group. LEfSe searched for the biomarker function of each group (LDA> threshold function, with higher abundance in the corresponding group and lower abundance in other groups). That is, functions that are significantly more abundant in this group than in the other groups.

### Statistical analysis

Descriptive statistics are presented as numbers and percentages for categorical variables, as means and standard deviations (SD) for continuous variables, and as medians of interquartile intervals (IQRs) if continuous variables are not normally distributed. Parametric tests (Student’s t-test and one-way ANOVA) and nonparametric tests (Mann-Whitney and Kruskal-Wallis tests) were used, as appropriate, taking into account the assumption of normality and the number of groups compared. Bonferroni tests were performed to correct for multiple testing. The Mann-Whitney test was used to measure the significance of metagenomic data. The Kolmogorov-Smirnov test was used to test the assumption of normality of the distribution of variables. Significant predictors of 60-day mortality (P<0.1) were used in a multiple regression model integrating odds ratio (OR) with 95% confidence interval (CI). The forward stepwise method was used, and the goodness of fit was tested by the Hosmer-Lemeshow test. For categorical variables, the chi-square test and Fisher’s exact test were used as appropriate. The Alpha diversity and beta diversity was measured with the principal coordinate analysis (PCoA) using Bray-Curtis and unweighted unifrac dissimilarity matrices. The dysbiosi index for the EN vs PN group was calculated using the selbal algorithm (in R-version 4.2.2), which takes into account the constitutive nature of the microbiota data (30, 31). The moderating effect of the association between the ecological imbalance index (as a continuous variable) and mortality was further assessed in a Cox regression, adjusting for covariates of differences in trend levels. Spearman correlation analysis was performed to determine the relationship between fecal microbiome abundance and clinical characteristics. The corrplot package is a graphical display of a correlation matrix (R-4.2.2 for Windows). All statistical analyses were conducted by using SPSS software (version 26.0), and statistical significance was defined as a P value<0.05.

### Functional analysis of metagenomic sequencing data

The non-redundant genes were functional annotated using the blastp function of Diamond (0.8.24) (32), and then the intestinal microbiota functional analysis was performed. Functional metabolic pathway enrichment analysis was performed using Kyoto Encyclopedia of Genes and Genomes (KEGG) and the cluster of orthologous groups of proteins (COG) database based on the predicted metagenome. In addition, Statistical Analysis of Metagenomic Profiles (STAMP) was employed to analyze the number of marker genes assigned to a functional profile indicating the number of sequences assigned to different biological subsystems or pathways (33). The predicted KEGG and COG pathways were compared between groups using STAMP, and the P value of significance (by factorial Kruskal-Wallis test) was 0.05.

## Results

### Clinical characteristics of elderly patients with COVID-19

**Table 1** presents the key attributes, clinical variables, and outcome measures associated with elderly individuals affected by COVID-19. The study encompassed a cohort of 71 older adults who were confirmed positive for SARS-CoV-2 through laboratory testing. The research was conducted between January 2023 and February 2023, and the participants were assigned to receive either EN or PN as a means of nutritional support. Within the EN group, there were 19 females and 29 males, with an average age of 77.67 ± 9.12 years. In contrast, the PN group comprised of 4 females and 19 males, with a mean age of 80.43 ± 8.29 years. There were no statistically significant differences in terms of age and gender between the two groups, as shown in **Table 1**. Regarding comorbidities, the EN group consisted of 17 patients (35.4%) with diabetes, 23 patients (47.9%) with hypertension, 21 patients (6.3%) with chronic respiratory disease, 6 patients (12.5%) with chronic cardiac disease, and 5 patients (10.4%) with a history of cancer, as indicated in **Table 1**. Additionally, the PN group demonstrated statistically significant reductions in PaO2/FiO2 ratio, respiratory index, and resting arterial oxygen saturation (P<0.05). Conversely, white blood cells (P=0.032), neutrophils (P=0.020), and procalcitonin (P<0.001) were significantly elevated in the PN group compared to the EN group. Furthermore, there was no significant difference in antibiotic exposure between the two groups, as 33 patients (68.8%) in the EN group received antibiotics during hospitalization, which was not significantly different from the PN group (P=0.099). A significant difference was not observed between the groups when it came to Covid-19-specific therapies such as steroids and intravenous immunoglobulins (**Table 2**). The patients in the PN group were significantly more likely to undergo ICU admission (P<0.001), require invasive ventilation (P<0.001), suffer respiratory failure, shock, complications, organ failure, and die within 60 days (P=0.001). In the present study, a cohort of 11 patients was admitted to the ICU exclusively from the PN group for therapeutic purposes. The duration of their stay in the ICU was recorded as 12.00 days [IQR, 8.00-32.00 days]. The comprehensive breakdown of complications in the elderly patients diagnosed with COVID-19 pneumonia can be found in **Table 3**.

**Table 1:** Baseline characteristics of patients infected with COVID-19 pneumonia.

	EN group(n=48)	PN group(n=23)	P-value
Age, years	77.67 ± 9.12	80.43 ± 8.29	0.222
Male gender (%)	29(60.4)	19(82.6)	0.061
BMI, kg/m^2^	23.14 ± 3.43	21.47 ± 3.69	0.065
Smoking (n, %)	2(4.2)	2(8.7)	0.591
Alcohol (n, %)	0(0.0)	1(4.3)	0.324
Diet (n, %)			0.759
Balanced	39(81.3)	18(78.3)	
Unbalanced	9(18.8)	5(21.7)	
**Symptoms at admission (n, %)**			
Cough	40(83.3)	19(82.6)	1.000
Sputum	21(43.8)	8(34.8)	0.472
Fever	33(68.8)	17(73.9)	0.656
Fatigue	5(10.4)	2(8.7)	1.000
Myalgia	10(20.8)	3(13.0)	0.526
Dyspnea	5(10.4)	3(13.0)	0.708
**Admission measures, mean (SD)**			
Respiratory rate, breaths/min	21.17 ± 2.47	21.43 ± 3.27	0.702
Heart rate, beats/min	88.88 ± 15.68	87.74 ± 22.59	0.806
Blood pressure, mm Hg			
Systolic	134.48 ± 18.94	124.91 ± 22.51	0.065
Diastolic	78.81 ± 11.56	73.61 ± 15.08	0.113
Arterial oxygen concentration (at rest)	96.96 ± 4.32	92.47 ± 9.06	**0.006**
PaO2/FiO2 (ratio)	334.66 ± 153.94	211.73 ± 115.43	**0.001**
Respiratory index	1.26 ± 1.35	2.86 ± 2.34	**<0.001**
**Location of CT findings**			0.162
Unilateral pneumonia	16(33.3)	4(17.4)	
Bilateral pneumonia	32(66.7)	19(82.6)	
**Oxygen therapy**			
Non-invasive ventilation	1(2.1)	1(4.3)	0.546
Invasive mechanical ventilation (n, %)	0(0.0)	12(52.2)	**<0.001**
**Comorbidities (n, %)**			
Hypertension	23(47.9)	10(43.5)	0.726
Diabetes mellitus II	17(35.4)	5(21.7)	0.243
Chronic cardiac disease	6(12.5)	5(21.7)	0.319
Chronic obstructive lung disease	3(6.3)	0(0.0)	0.546
Cancer	5(10.4)	1(4.3)	0.656
**Laboratory parameters**			
Haemoglobin, g/dL	123.23 ± 22.09	110.83 ± 28.91	0.050
Albumin, g/L	33.84 ± 4.97	30.45 ± 5.07	**0.009**
C–reactive protein, mg/L	59.41 ± 70.74	93.13 ± 59.56	0.052
Red blood cell count	4.00 ± 0.67	3.90 ± 1.00	0.625
White blood cell count	7.69 ± 3.83	10.25 ± 5.95	**0.032**
Neutrophil cell count, ×10^9^/L	5.68 ± 3.74	8.26 ± 5.29	**0.020**
Lymphocyte count, ×10^9^/L	1.29 ± 0.67	1.36 ± 2.16	0.829
Platelet count, ×10^9^/L	254.75 ± 119.81	236.65 ± 113.18	0.546
Creatinine, μmol/L	85.83 ± 34.38	90.13 ± 52.54	0.681
Total bilirubin, mmol/L	13.13 ± 6.67	14.92 ± 8.36	0.360
Aminotransferase, U/L			
Alanine	25.49 ± 16.92	29.54 ± 23.25	0.409
Aspartate	34.52 ± 26.80	36.37 ± 27.48	0.788
D-dimer level, mg/L	2.89 ± 7.85	4.55 ± 7.64	0.403
Lactate dehydrogenase, U/L	252.79 ± 119.51	286.30 ± 133.91	0.308
Procalcitonin, ng/mL			**<0.001**
Normal	41(85.4)	10(43.5)	
Elevated	7(14.6)	13(56.5)	

The meaning of the values in bold is the P-value is less than 0.05.

**Table 2:** Comparison of clinical outcomes of different nutritional support therapies in patients with COVID-19.

	EN group(n=48)	PN group(n=23)	P-value
**Therapeutic management of COVID-19**			
Antibiotics (n, %)	33(68.8)	20(87.0)	0.099
Dexamethasone (n, %)	18(37.5)	11(47.8)	0.407
Intravenous immunoglobulins (n, %)	22(45.8)	12(52.2)	0.617
**Complications during hospitalization (n, %)**	5(10.4)	16(69.6)	**<0.001**
**Outcomes**			
Respiratory failure (n, %)	5(10.4)	13(56.5)	**<0.001**
Shock (n, %)	0(0.0)	11(47.8)	**<0.001**
Organ failure (n, %)	1(2.1)	13(56.5)	**<0.001**
Use vasopressors (n, %)	0(0.0)	12(52.2)	**<0.001**
Intensive care unit admission (n, %)	0(0.0)	11(47.8)	**<0.001**
60-day mortality	1(2.1)	7(30.4)	**0.001**

The meaning of the values in bold is the P-value is less than 0.05.

**Table 3:** Details of complications in patients with COVID-19 pneumonia.

	EN group (n=5)	PN group (n=16)
Acute respiratory failure	0	4
Pleural effusion	2	2
Gastrointestinal hemorrhage	0	1
Venous thrombosis	2	0
Acute kidney injury	0	4
Urinary tract infection	0	1
Acute pulmonary embolism	0	1
Abnormal liver function	0	1
Hypokalemia	0	1
Hypoproteinemia	1	1

The 60-day mortality rate was found to be 2.1% (1/48) in the EN group and 30.4% (7/23) in the PN group (P=0.001). To determine the independent predictors of 60-day mortality, a stepwise logistic regression analysis was conducted, incorporating age (as a continuous variable), sex (male or female), presence of concomitant diseases (yes or no), and body mass index (also continuous) as variables. Additionally, univariate logistic regression analysis revealed that nine other variables significantly influenced the 60-day mortality rate. The variables included in the analysis were nutrition type (enteral, parenteral), systolic blood pressure, diastolic blood pressure, respiratory index, invasive ventilation (no, yes), hemoglobin count (continuous), white blood cell count (continuous), neutrophil count (continuous), and creatinine count (continuous). Consequently, these parameters were also incorporated into the multiple regression analysis. To assess the adequacy of the logistic regression model, the Hosmer-Lemeshow statistic was employed. Multivariate regression analysis showed that high respiratory index [odds ratio (OR): 1.835, 95% confidence interval (CI): 1.181-2.851], invasive respiration [OR: 0.012, 95% CI: 0.001-0.121], low hemoglobin [OR: 0.947, 95%CI: 0.911-0.984], and PN support [OR: 0.049, 95%CI: 0.006-0.426] was a predictor of mortality. These models are both illustrated in **Table 4**. To rule out the possibility that the choice of nutritional pattern is based on disease severity, we have established a multiple regression model that takes into account all confounding factors that may affect disease severity. Our results showed that the choice of nutritional approach was not significantly associated with the severity of illness at admission, but was associated with organ failure and complications when using regression analysis with baseline information at admission (**Table 5**).

**Table 4:** Multivariate logistic regression analysis of clinical variables associated with 60-day mortality in patients with COVID-19.

	Survivors (n=63)	60-day mortality (n=8)	P value	Multivariate		
				Odds Ratio	95% Confidence Interval	P value
Age, years	78.25 ± 8.85	81.00 ± 9.46	0.415			
Male gender (%)	43(68.3)	5(62.5)	0.708			
BMI, kg/m^2^	22.56 ± 3.58	22.81 ± 3.77	0.851			
Smoking (n, %)	3(4.8)	1(12.5)	0.387			
Alcohol (n, %)	1(1.6)	0(0.0)	1.000			
**Nutritional mode**			**0.001**	0.049	0.006-0.426	**0.006**
Enteral nutrition	47(74.6)	1(12.5)				
Parenteral nutrition	16(25.4)	7(87.5)				
**Admission measures, mean (SD)**						
Respiratory rate, breaths/min	21.17 ± 2.66	21.88 ± 3.44	0.499			
Heart rate, beats/min	88.54 ± 17.98	88.25 ± 19.83	0.966			
Blood pressure, mm Hg						
Systolic	133.62 ± 18.69	113.75 ± 26.63	**0.009**			0.163
Diastolic	78.49 ± 12.48	66.38 ± 12.09	**0.012**			0.284
Arterial oxygen concentration (at rest)	95.69 ± 6.58	94.00 ± 6.47	0.494			
PaO2/FiO2 (ratio)	305.36 ± 150.84	212.00 ± 155.54	0.105			
Respiratory index	1.55 ± 1.65	3.55 ± 2.63	**0.004**	1.835	1.181-2.851	**0.007**
**Location of CT findings**			0.427			
Unilateral pneumonia	19(30.2)	1(12.5)				
Bilateral pneumonia	44(69.8)	7(87.5)				
**Oxygen therapy**						
Non-invasive ventilation	2(3.2)	0(0.0)	1.000			
Invasive mechanical ventilation (n, %)	5(7.9)	7(87.5)	**<0.001**	0.012	0.001-0.121	**<0.001**
**Comorbidities (n, %)**						
Hypertension	28(44.4)	5(62.5)	0.459			
Diabetes mellitus II	19(30.2)	3(37.5)	0.696			
Chronic cardiac disease	8(12.7)	3(37.5)	0.101			
Chronic obstructive lung disease	3(4.8)	0(0.0)	1.000			
Cancer	6(9.5)	0(0.0)	1.000			
**Laboratory parameters**						
Haemoglobin, g/dL	122.46 ± 23.12	93.63 ± 25.89	**0.002**	0.947	0.911-0.984	**0.006**
Albumin, g/L	33.13 ± 5.21	29.69 ± 4.43	0.080			
C–reactive protein, mg/L	68.09 ± 68.31	88.05 ± 74.43	0.443			
Red blood cell count	4.01 ± 0.75	3.63 ± 0.99	0.201			
White blood cell count	8.03 ± 3.99	12.37 ± 7.98	**0.013**			0.190
Neutrophil cell count, ×10^9^/L	5.94 ± 3.56	11.01 ± 7.65	**0.002**			0.118
Lymphocyte count, ×10^9^/L	1.38 ± 1.39	0.78 ± 0.45	0.239			
Platelet count, ×10^9^/L	247.89 ± 115.32	256.75 ± 139.66	0.842			
Creatinine, μmol/L	82.48 ± 30.46	124.54 ± 81.49	**0.005**			0.064
Total bilirubin, mmol/L	14.14 ± 7.59	10.84 ± 3.23	0.233			
Aminotransferase, U/L						
Alanine	26.78 ± 17.85	27.08 ± 28.96	0.967			
Aspartate	35.41 ± 26.44	32.79 ± 31.73	0.797			
D-dimer level, mg/L	3.28 ± 7.87	4.55 ± 7.35	0.667			
Lactate dehydrogenase, U/L	257.88 ± 128.06	308.61 ± 89.78	0.284			
Procalcitonin, ng/mL			0.209			
Normal	47(74.6)	4(50.0)				
Elevated	16(25.4)	4(50.0)				
Respiratory failure (n, %)	5(10.4)	13(56.5)	**<0.001**			0.383
Shock (n, %)	0(0.0)	11(47.8)	**<0.001**	0.797	0.372-1/750	**<0.001**
Organ failure (n, %)	1(2.1)	13(56.5)	**<0.001**			0.817
Use vasopressors (n, %)	0(0.0)	12(52.2)	**<0.001**			0.896
Intensive care unit admission (n, %)	0(0.0)	11(47.8)	**<0.001**			0.709

The meaning of the values in bold is the P-value is less than 0.05.

**Table 5:** Multivariate logistic regression analysis of clinical variables associated with disease severity in patients with COVID-19.

	Enteral nutrition group(n=48)	Parenteral nutrition group(n=23)	P value	Multivariate
				P value
Arterial oxygen concentration (at rest)	96.96 ± 4.32	92.47 ± 9.06	**0.006**	0.958
PaO2/FiO2 (ratio)	334.66 ± 153.94	211.73 ± 115.43	**0.001**	0.856
Respiratory index	1.26 ± 1.35	2.86 ± 2.34	**<0.001**	0.592
**Comorbidities (n, %)**				
Hypertension	23(47.9)	10(43.5)	0.726	0.571
Diabetes mellitus II	17(35.4)	5(21.7)	0.243	0.101
Chronic cardiac disease	6(12.5)	5(21.7)	0.319	0.446
Chronic obstructive lung disease	3(6.3)	0(0.0)	0.546	0.552
Cancer	5(10.4)	1(4.3)	0.656	0.105
Albumin, g/L	33.84 ± 4.97	30.45 ± 5.07	**0.009**	0.326
White blood cell count	7.69 ± 3.83	10.25 ± 5.95	**0.032**	0.626
Neutrophil cell count, ×10^9^/L	5.68 ± 3.74	8.26 ± 5.29	**0.020**	0.832
Procalcitonin, ng/mL			**<0.001**	0.109
Normal	41(85.4)	10(43.5)		
Elevated	7(14.6)	13(56.5)		
**Complications during hospitalization (n, %)**	5(10.4)	16(69.6)	**<0.001**	**<0.001**
**Outcomes**				
Respiratory failure (n, %)	5(10.4)	13(56.5)	**<0.001**	0.847
Shock (n, %)	0(0.0)	11(47.8)	**<0.001**	0.251
Organ failure (n, %)	1(2.1)	13(56.5)	**<0.001**	**<0.001**

The meaning of the values in bold is the P-value is less than 0.05.

### Changes of gut microbiota in elderly patients with COVID-19 under different nutritional support

**Figure 2:**
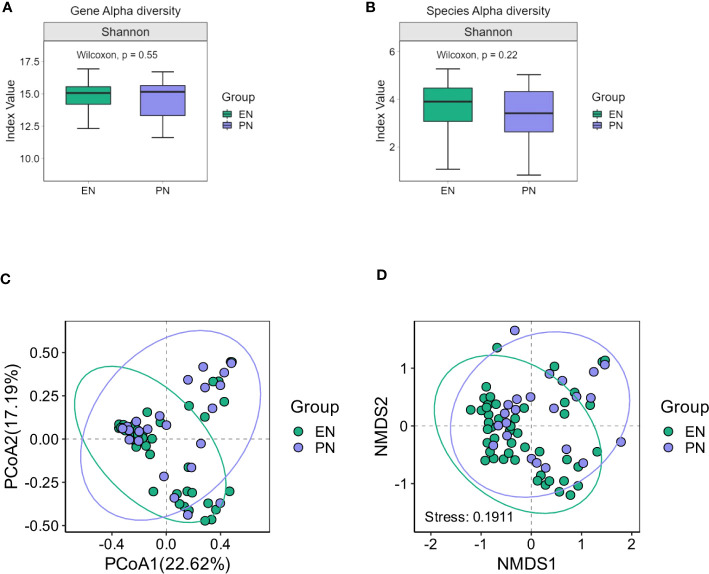
Changes of gut microbiota in the EN group and PN group. **(A)** Alpha diversity of gut microbiota genes between the EN group and PN group, **(B)** Alpha diversity of gut microbiota species between the EN group and PN group, **(C)** The β diversity of gut microbiota in the EN group and PN group was illustrated using PCoA diagrams, **(D)** The β diversity of gut microbiota in the EN group and PN group was illustrated using NMDS plots.

The presence of SARS-CoV-2 viral RNA in the gastrointestinal tract leads to infection of intestinal cells and the duodenum, indicating its impact on the gut microbiome’s composition. To evaluate this, 16S ribosomal RNA (rRNA) gene analysis was conducted on 71 fecal samples to determine the microbial composition. Changes in the relative abundance (median) of each bacterial genus were assessed to examine alterations in the gut microbiota composition among COVID-19 patients in both the PN and EN groups. Notably, no significant difference in bacterial gene alpha diversity was observed between the PN and EN groups, as depicted in **Figure 2A**. Next, we proceeded to analyze the Alpha diversity of the species in both the EN group and PN group. The Chao1, Shannon, and Simpson indices were employed as commonly utilized measures of Alpha diversity. Notably, the analysis of the Shannon diversity index revealed no statistically significant difference between the two groups (P>0.05, Wilcoxon rank sum test) (**Figure 2B**).

**Figure 3:**
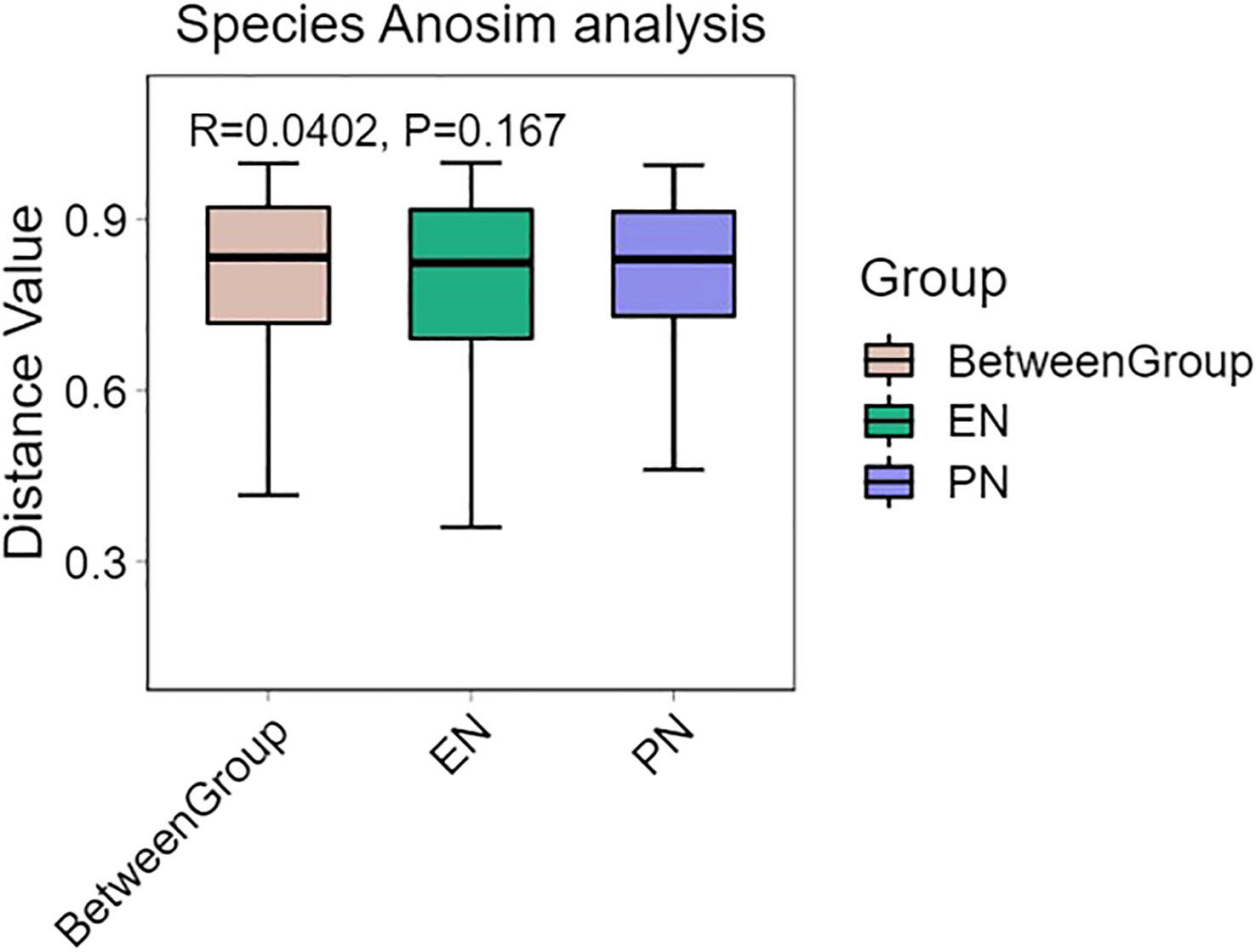
Analysis of similarities of gut microbiota between the EN group and PN group.

**Figure 4:**
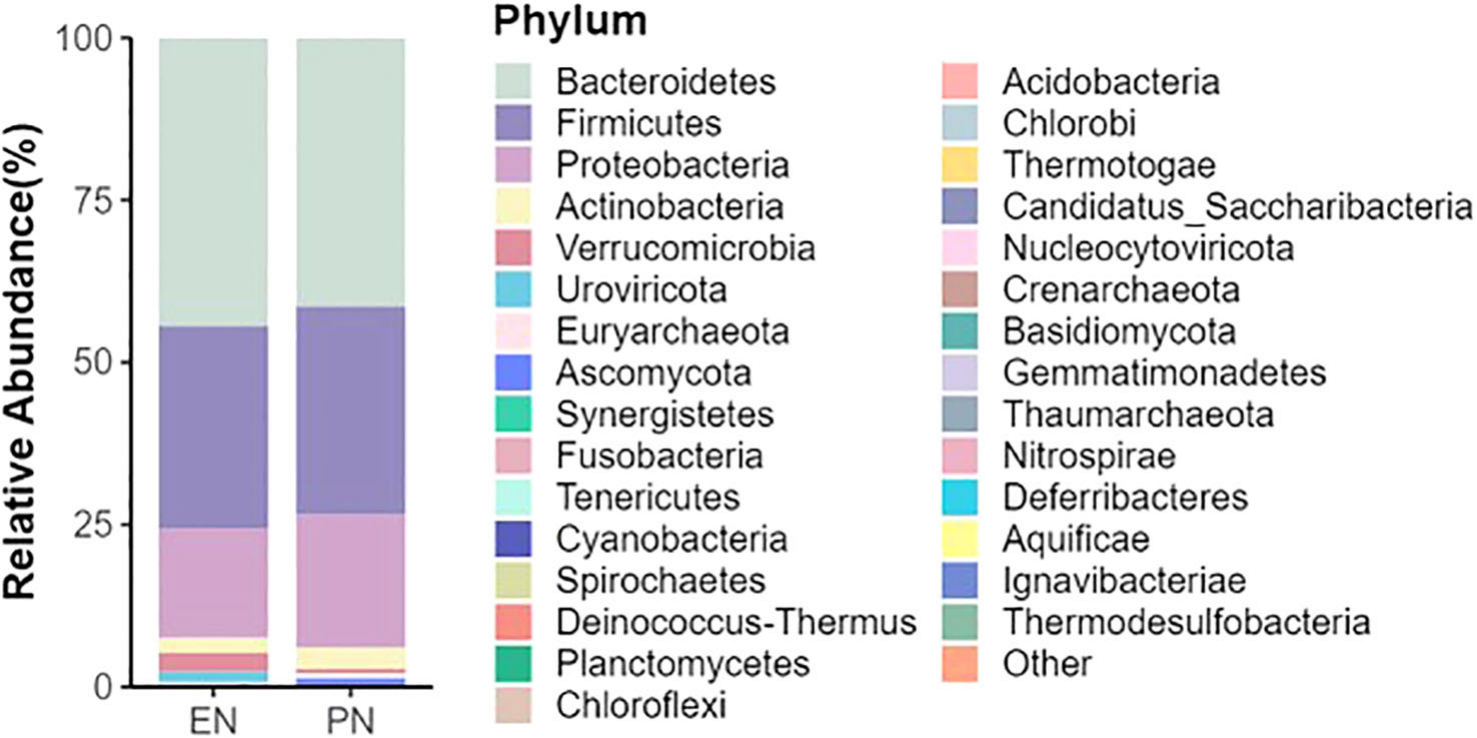
Relative abundance of gut microbiota on phylum in the EN group and PN group.

**Figure 5:**
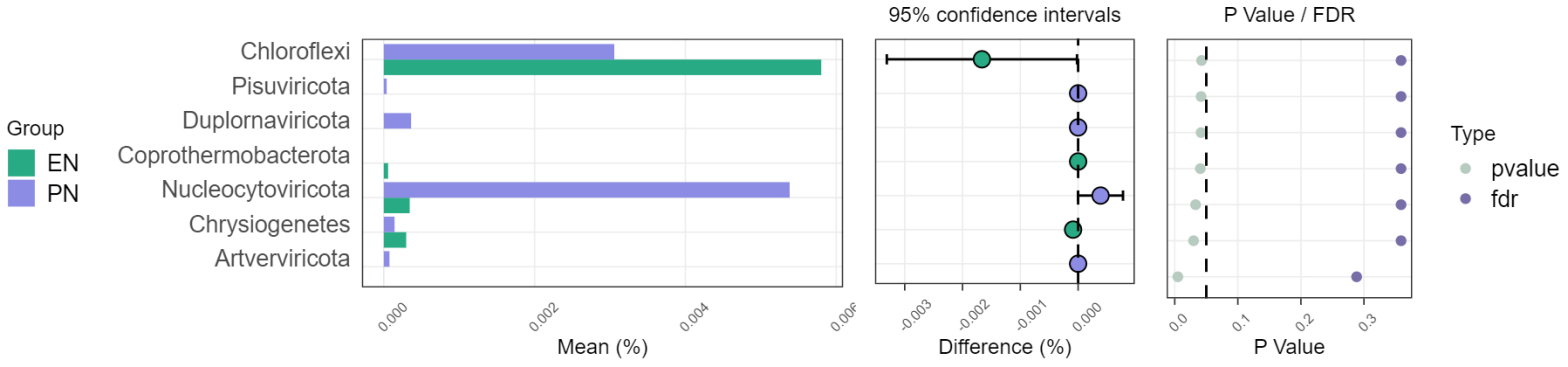
Comparison of the relative abundance of the gut microbiota on the phylum between the EN group and PN group.

**Figure 6:**
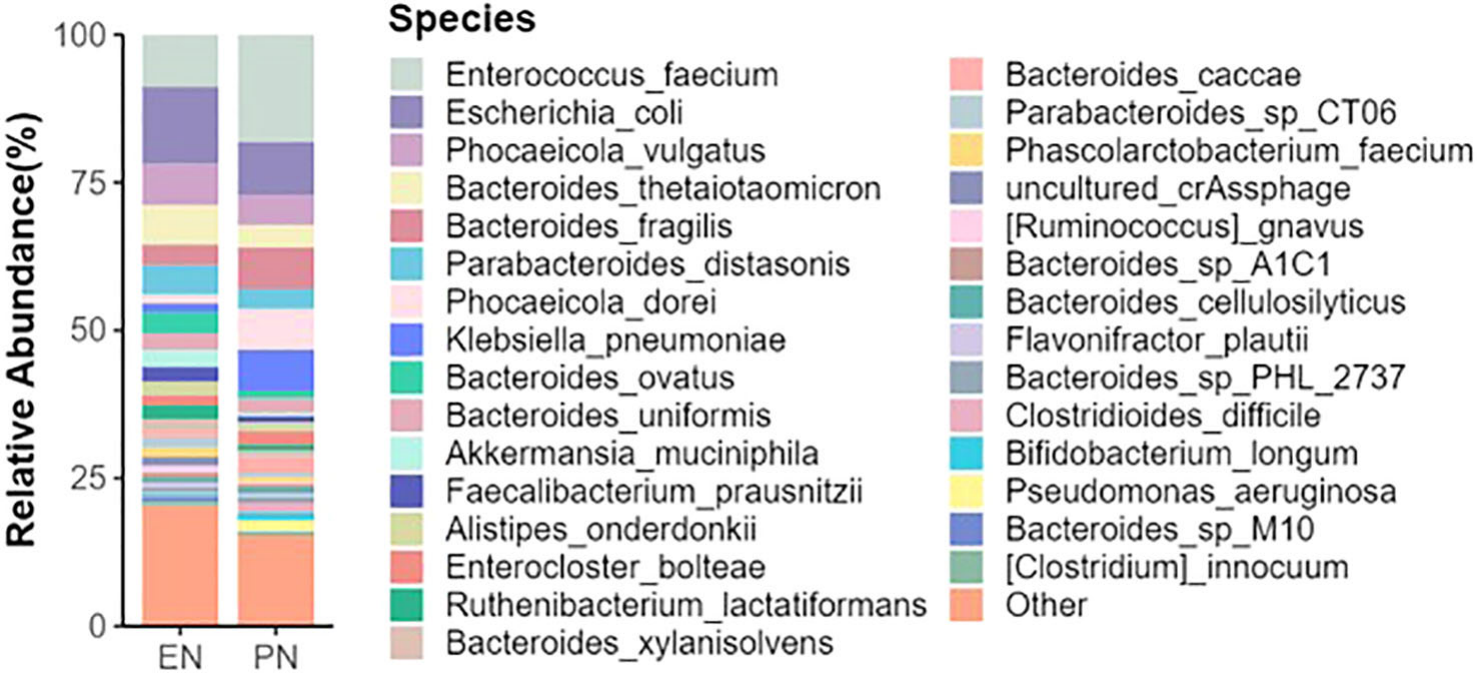
Relative abundance of gut microbiota species in the EN group and PN group.

To ascertain potential dissimilarities in the overall gut microbial composition between the two groups, we investigated various markers of beta diversity. The differences in fecal microbial communities between the two groups (EN and PN) were visualized using Intestinal microbiota PCoA and Non-metric multi-dimensional (NMDS) based on Bray-Curtis difference scaling (**Figures 2C, D**). Additionally, Analysis of similarities (Anosim) was employed to examine the dissimilarities between the two groups. The findings indicated that while there were resemblances between the two groups, no statistically significant differences were observed (P=0.167) (**Figure 3**). Subsequently, an examination was conducted to assess the alterations in the relative abundance of intestinal microflora among COVID-19 patients receiving varying forms of nutritional support at the phylum level. Specifically, the two patient cohorts primarily consisted of *Bacteroidetes*, *Firmicutes*, and *Proteobacteria* at this taxonomic level. Notably, these distinctions did not reach statistical significance (**Figure 4**). However, employing STAMP analysis, it was observed that the EN group exhibited a higher relative abundance of Chloroflexi member in comparison to the PN group among elderly COVID-19 individuals (mean 0.58% vs 0.31%, P=0.043). In COVID-19 individuals, the abundance of *Nucleocytoviricota* members in the PN group was significantly higher compared to the EN group (mean 0.34% vs. 0.54%, P=0.033) (**Figure 5**). Regarding bacterial composition, *Enterococcus_faecium, Escherichia_coli, Phocaeicola_vulgatus, Bacteroides_thetaiotaomicron, Bacteroides_fragilis*, and *Bacteroides_Fragilis* were identified as the most prevalent bacterial genera in both groups (**Figure 6**).

**Figure 7:**
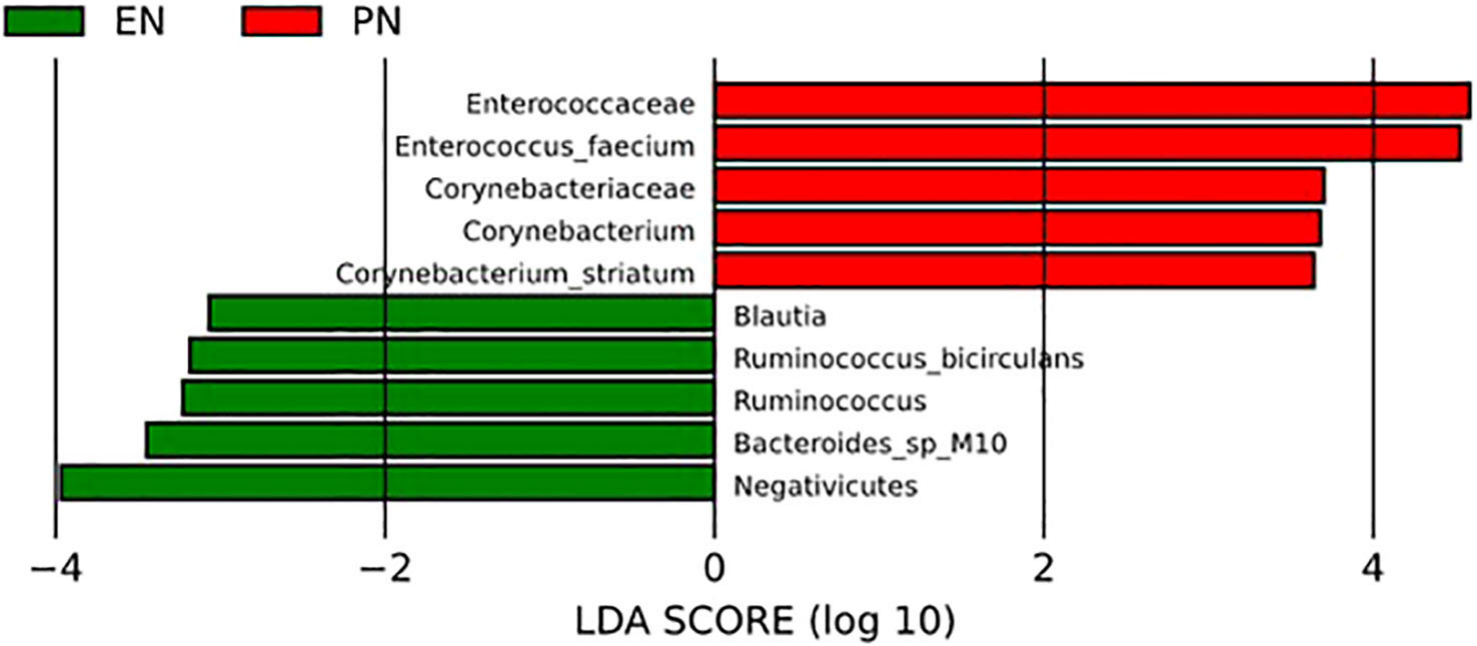
LEfSe analysis was used to compare the relative abundance of gut microbiota species between the EN group and PN group.

To further examine alterations in bacterial abundance, the researchers employed linear discriminant analysis effect size (LEfSe) to compare the variations in gut microbiota between the two groups of COVID-19 patients. The LEfSe analysis successfully identified several taxa at various levels, which exhibited dissimilarities among elderly COVID-19 patients receiving distinct nutritional support therapies. LEfSe feature selection using LDA scores greater than 3.0 successfully identified a number of bacterial genera that exhibited discriminatory characteristics between subjects in the EN group and PN group. Notably, the elderly patients with COVID-19 in the PN group demonstrated a significant increase in *Enterococcus faecium*, *Pseudomonas aeruginosa*, and *Corynebacterium* striatum compared to patients in the EN group. Conversely, the EN group exhibited significant increases in *Ruminococcus, Negativicutes, Bacteroides*, and *Blautia* (**Figure 7**).

**Figure 8:**
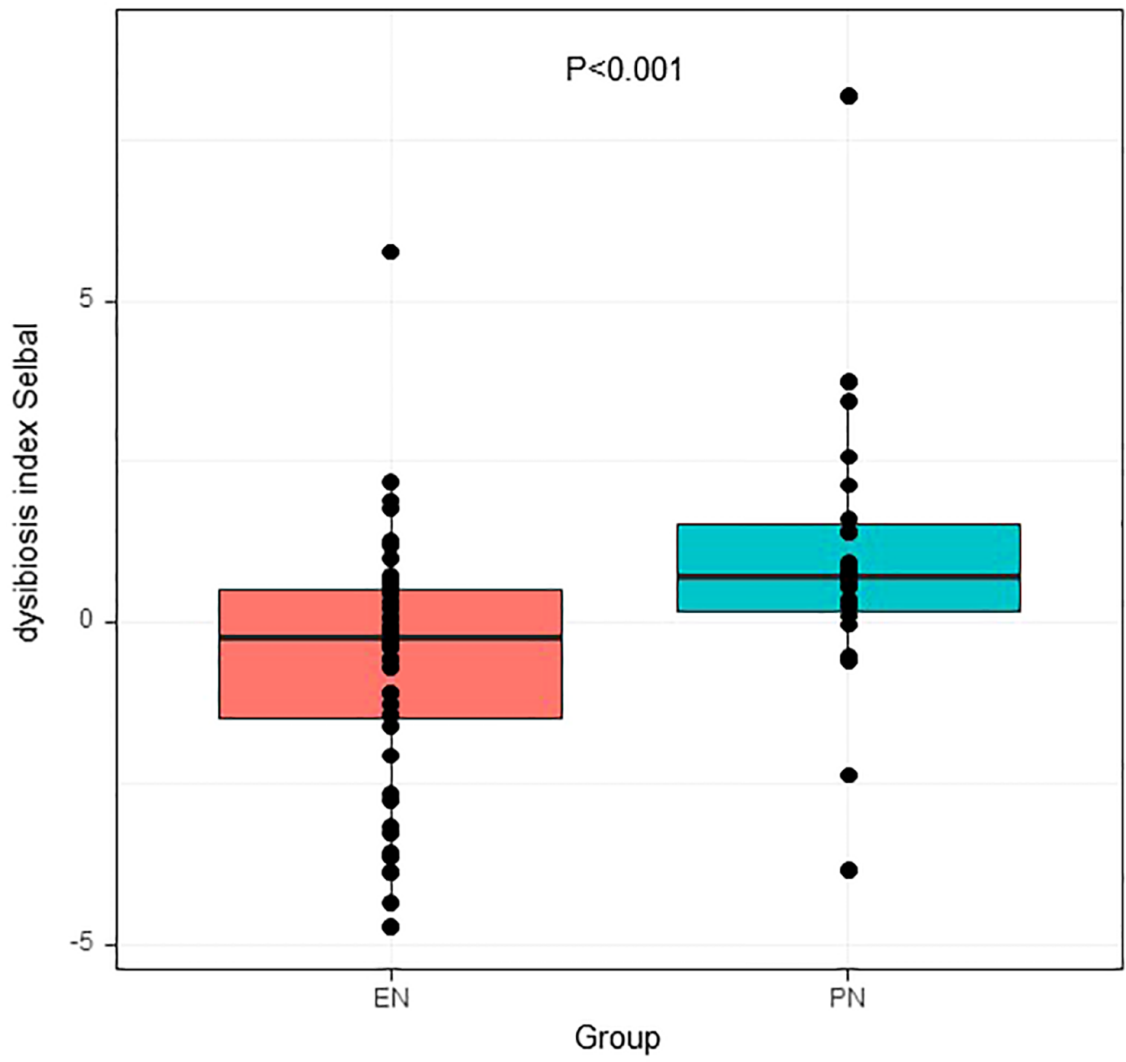
P values of dysbiosis index between patients in the EN group and PN group.

**Figure 9:**
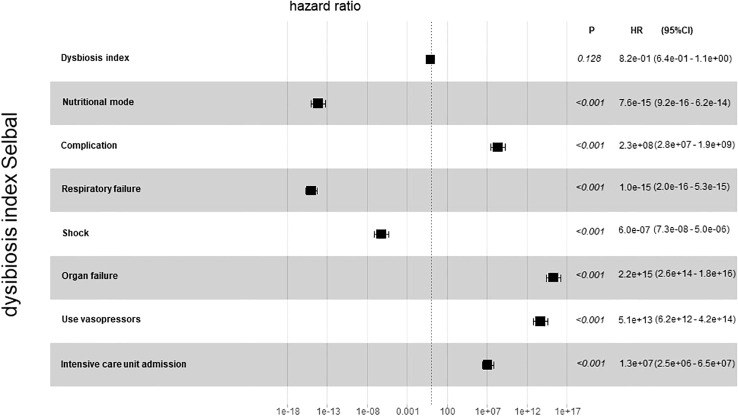
Cox regression analysis for dysbiosis index with covariates that show significant differences between the EN group and PN group.

For further analysis, we generated the dysbiosis index for EN vs PN group based on the R package selbal, which was used to select bacterial taxa that differentiated patients in the EN group from those in the PN group. This index is defined as the difference between arithmetic means of log-transformed abundances in these two groups of taxa (30, 31). As shown in **Figure 8**, this index was associated with nutritional support therapy (P<0.001). We further performed Cox regression analysis of the dysbiosis index with the covariates that showed significant differences between the EN group and PN group in **Table 2**. The results show that the clinical outcomes of patients in the EN group and the PN group, and separately adjusting for markers with different trend levels between the two groups did not change this association (**Figure 9**). To further analyze the relationship between gut microbiota and related confounding factors in elderly COVID-19 patients, including the use of antibiotics or other drugs, we correlated the dysbiosis index of EN vs PN group with baseline data. We further grouped elderly COVID-19 patients with or without antibiotic or dexamethasone treatment and calculated the relationship with the ecological disequilibrium index. Our results showed that patients receiving antibiotic and immunoglobulin treatment had higher ecological disequilibrium indexes, while patients receiving dexamethasone treatment had lower ecological disequilibrium indexes, but without statistical difference (P>0.05) (**Table 6**). In addition, there was no significant correlation between the ecological imbalance index and the severity of disease in patients (**Table 7**).

**Table 6:** Effects of drug therapy on dysbiosis index in hospitalized elderly patients with COVID-19.

	Dysbiosis index		
	No treatment	Treatment	P value
	No treatment	Treatment	P value
Antibiotics	-0.589 ± 0.482	0.090 ± 0.306	0.259
Dexamethasone	0.108 ± 0.344	-0.357 ± 0.398	0.383
Intravenous immunoglobulins	-0.424 ± 0.304	0.290 ± 0.426	0.172

**Table 7:** Correlation analysis of baseline and ecological index of COVID-19.

	Dysbiosis index	
	*Pearson’s r*	P-value
Arterial oxygen concentration (at rest)	-0.224	0.060
PaO2/FiO2 (ratio)	-0.218	0.067
Respiratory index	0.223	0.061
Albumin, g/L	-0.211	0.077
White blood cell count	0.167	0.164
Neutrophil cell count	0.212	0.075
Procalcitonin	0.212	0.076

### Relationship between gut microbial composition and clinical indicators

**Figure 10:**
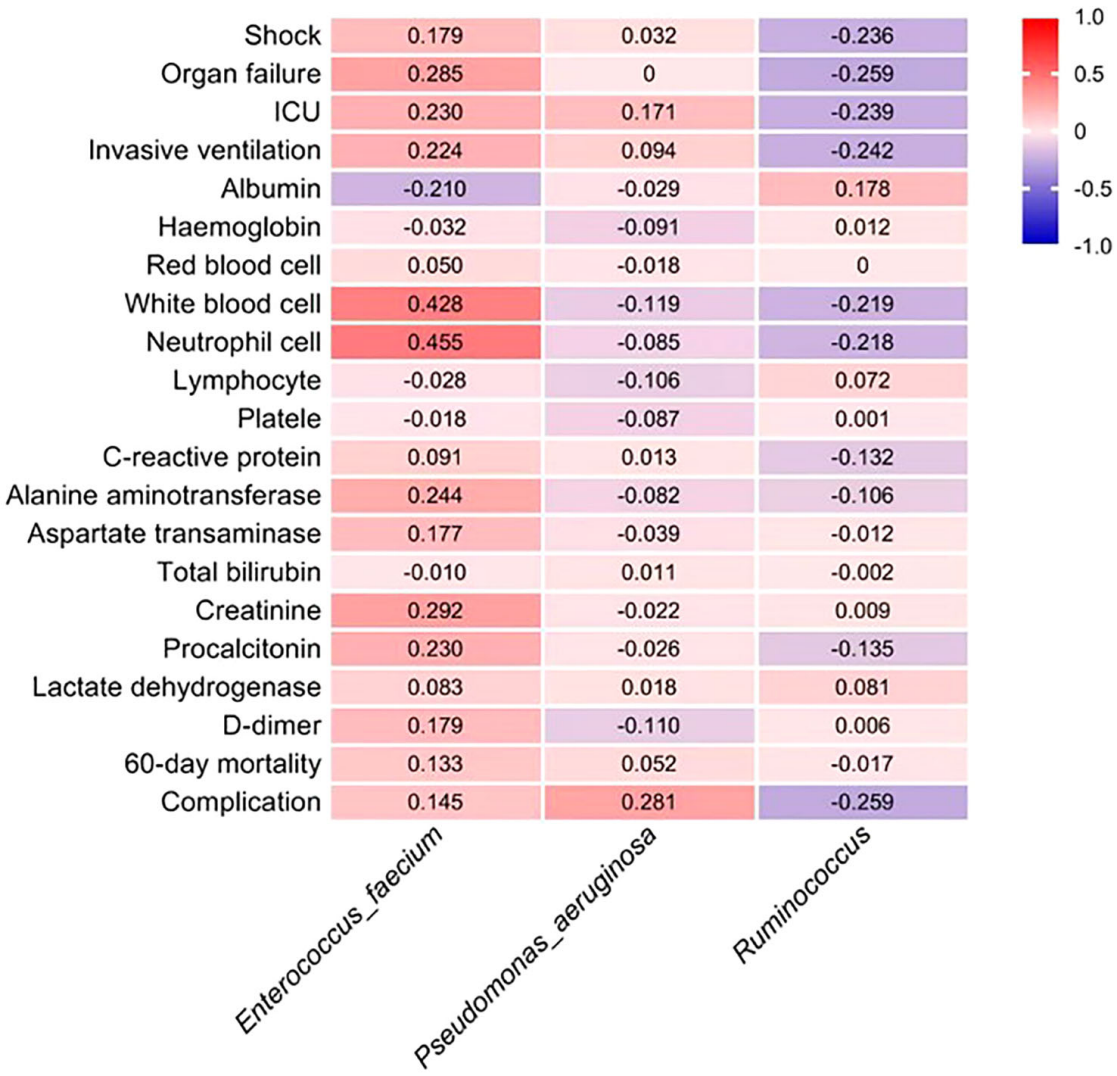
The estimated Spearman’s r values, as shown in the heat map, were obtained by correlation analysis between the phase abundance of differential gut microbiota species between the two groups and the values of clinical prognostic parameters. Red and dark blue indicate positive and negative correlations between the two color, respectively.

The LEfSe analysis was employed to assess the disparity in gut microbiota between the two cohorts of COVID-19 patients. In comparison to the EN group, the PN group exhibited a noteworthy elevation in the relative abundance of *Enterococcus_faecium* and *Pseudomonas_aeruginosa*, whereas the relative abundance of *Ruminococcus* was significantly diminished. To ascertain the potential association between the distinct microbial compositions in the two cohorts and the clinical indicators, we conducted a Spearman correlation analysis. This analysis aimed to investigate the interplay between the gut microbiota and the clinical indicators in both groups of COVID-19 patients. The findings revealed a positive correlation between *Enterococcus_faecium* and Organ failure (r=0.285, P=0.016), as well as White blood cell parameter (r=0.428, P<0.001). The Neutrophil cell parameter (r=0.455, P<0.001), Alanine aminotransferase parameter (r=0.244, P=0.042), and Creatinine parameter (r=0.292, P=0.013) exhibited positive correlations. Additionally, the genus *Pseudomonas_aeruginosa* demonstrated a positive correlation with complications during hospitalization (r=0.281, P=0.018). Furthermore, the presence of *Ruminococcus* exhibited a negative correlation with the incidence of Shock (r=-0.236, P=0.048), Organ failure (r=-0.259, P=0.029), and ICU transition (r=-0.239, P=0.044). Additionally, it displayed a negative correlation with the Invasive ventilation parameter (r=-0.242, P=0.042) and complications during hospitalization (r=-0.259, P=0.029) as indicated in **Table 8**. To visually represent the relationship between the relative abundance of three distinct flora and each clinical indicator, a heat map was employed (**Figure 10**).

**Table 8:** Correlation analysis between clinical prognosis and relative abundance of differential flora of COVID-19.

		*Enterococcus_faecium*	*Pseudomonas_aeruginosa*	*Ruminococcus*
Shock	*Spearman’s r*	0.179	0.032	-0.236
	*P*	0.136	0.789	**0.048**
Organ failure	*Spearman’s r*	0.285	0.000	-0.259
	*P*	**0.016**	>0.999	**0.029**
ICU	*Spearman’s r*	0.230	0.171	-0.239
	*P*	0.054	0.154	**0.044**
Invasive ventilation	*Spearman’s r*	0.224	0.094	-0.242
	*P*	0.061	0.438	**0.042**
Albumin	*Spearman’s r*	-0.210	-0.029	0.178
	*P*	0.079	0.81	0.138
Haemoglobin	*Spearman’s r*	-0.032	-0.091	0.012
	*P*	0.789	0.448	0.920
Red blood cell	*Spearman’s r*	0.050	-0.018	0.000
	*P*	0.678	0.884	0.998
White blood cell	*Spearman’s r*	0.428	-0.119	-0.219
	*P*	**<0.001**	0.323	0.066
Neutrophil cell	*Spearman’s r*	0.455	-0.085	-0.218
	*P*	**<0.001**	0.481	0.068
Lymphocyte	*Spearman’s r*	-0.028	-0.106	0.072
	*P*	0.816	0.379	0.548
Platele	*Spearman’s r*	-0.018	-0.087	0.001
	*P*	0.883	0.471	0.992
C–reactive protein	*Spearman’s r*	0.091	0.013	-0.132
	*P*	0.450	0.915	0.273
Alanine aminotransferase	*Spearman’s r*	0.244	-0.082	-0.106
	*P*	**0.042**	0.498	0.384
Aspartate transaminase	*Spearman’s r*	0.177	-0.039	-0.012
	*P*	0.140	0.745	0.923
Total bilirubin	*Spearman’s r*	-0.010	0.011	-0.002
	*P*	0.936	0.931	0.985
Creatinine	*Spearman’s r*	0.292	-0.022	0.009
	*P*	**0.013**	0.859	0.937
Procalcitonin	*Spearman’s r*	0.230	-0.026	-0.135
	*P*	0.329	0.912	0.569
Lactate dehydrogenase	*Spearman’s r*	0.083	0.018	0.081
	*P*	0.513	0.888	0.520
D-dimer	*Spearman’s r*	0.179	-0.110	0.006
	*P*	0.134	0.359	0.963
60-day mortality	*Spearman’s r*	0.133	0.052	-0.017
	*P*	0.270	0.666	0.886
Complication	*Spearman’s r*	0.145	0.281	-0.259
	*P*	0.227	**0.018**	**0.029**

The meaning of the values in bold is the P-value is less than 0.05.

### Microbiota dysbiosis is associated with changes in metabolic function

**Figure 11:**
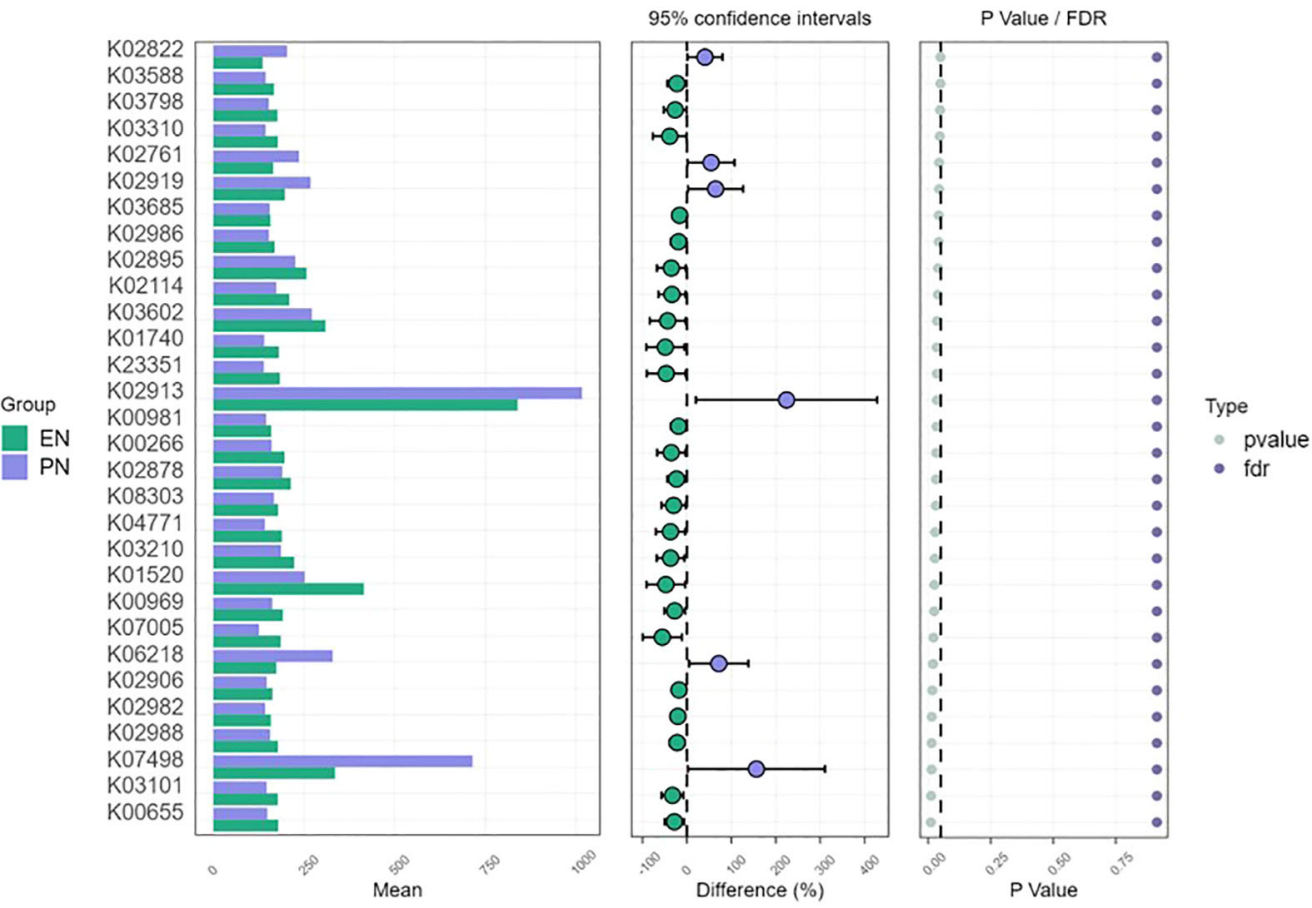
Metagenomic function prediction based on KEGG pathway analysis. Extended error bar plots of 30 different KEGG pathways through free filtering in patients in the EN group and PN group. Error bars showed significant differences in metabolic pathways between patients in the EN group and those in the PN group (P< 0.05, Kruskal Wallis test). The difference in the relative abundance of KEGG pathways was negative in the EN group and positive in the PN group.

The perturbation of the metabolic activity of the gut microbiota serves as an indicator of the severity of disease in individuals affected by COVID-19. Additionally, alterations in the relative prevalence of significant anticipated genomic functions were observed between the enteral and PN groups within the gut microbiota. Notably, the metabolic signatures identified within the same KEGG pathway exhibited variations across samples from patients receiving distinct nutritional support modalities, implying a potential correlation with early colonization and the progression of the disease. Changes in the metabolic profile and energy generation of immune cells have the potential to impact their activation, consequently worsening the imbalanced immune response observed in individuals with COVID-19. Additionally, we conducted STAMP analysis to identify functional disparities between the studied groups. Notably, genes responsible for ribosome synthesis, starch and sucrose metabolism, phosphotransferase system, ascorbate and uronate metabolism, as well as microbial metabolism in diverse environments, exhibited higher expression levels in the intestinal microbiome of patients in the PN group when compared to those in the EN group (P<0.05, **Figure 11**).

## Discussion

The prognosis of elderly patients with COVID-19 is significantly influenced by their nutritional status, making nutritional support a crucial component of their treatment (13). However, current nutritional therapy for COVID-19 patients primarily focuses on energy intake, feeding tube placement, nitrogen balance, and similar factors, neglecting the intricate interactions between nutrients and the host and the subsequent effects. The gut microbiota, a complex ecosystem within the host, plays a pivotal role in overall host health, and alterations in gut microbiota resulting from environmental influences can have significant implications (34). This study aimed to investigate the potential impact of gut microbiota dysbiosis on the pathophysiology and clinical prognosis of COVID-19 in elderly patients with various nutritional support modalities. Specifically, we conducted a prospective observational study involving 71 elderly COVID-19 patients, with the objective of examining the association between the presence of different diseases and the stability of the normal intestinal environment, which is considered a significant environmental factor. In order to investigate the correlation between the composition of gut microbiota and clinical outcomes in elderly individuals afflicted with COVID-19, who were subjected to varying nutritional regimens, an analysis was conducted on clinical data and 16S rRNA gene sequencing data obtained from COVID-19 patients. The findings of our study indicate that EN support yields a more favorable clinical prognosis for elderly COVID-19 patients. In comparison to PN support, EN support notably diminishes the occurrence of complications during hospitalization, organ failure, respiratory failure, as well as mortality within a span of 60 days. Furthermore, a multivariate regression analysis was employed to ascertain the autonomous factors that impact the 60-day mortality rate in elderly individuals afflicted with COVID-19. Notably, the nutritional pattern emerged as a distinct autonomous factor. Subsequently, an in-depth examination of the dissimilarities in gut microbiota between the two cohorts was conducted, revealing a significant elevation of *Enterococcus faecium*, Pseudomonas aeruginosa, and Corynebacterium striatum in elderly COVID-19 patients belonging to the PN group. However, the presence of *Ruminococcus* was notably higher in elderly COVID-19 patients belonging to the EN group. Subsequently, an examination of the relationship between distinct microbial communities and clinical prognosis was conducted. The analysis revealed a positive correlation between the presence of *Enterococcus faecium*, a specific microbial community, in elderly COVID-19 patients belonging to the PN group and clinical indicators of unfavorable prognosis. Conversely, the presence of *Ruminococcus* in elderly COVID-19 patients belonging to the EN group exhibited a negative correlation with clinical indicators of unfavorable prognosis.

Currently, research examining the immune response of the host has identified several features of COVID-19 cases, including cytokine storm, inadequate T cell response, lymphopenia, and aberrant activation of macrophages/monocytes (35). Nevertheless, the relationship between the host and microbiome throughout the course of COVID-19 remains uncertain. Gut microorganisms have the capacity to engage with the host immune system *via* direct physical interaction, metabolites, and alterations in the structural constituents of the intestinal mucosa (36).

In accordance with prior research, our study demonstrated an elevated presence of *Enterococcus* in elderly COVID-19 patients undergoing PN. Previous studies have indicated that *Enterococcus*, as a facultative pathogen, exhibits higher prevalence in severe cases of COVID-19 compared to mild cases (15, 37). Specifically, *Enterococcus faecium* is found to be enriched in severe COVID-19 cases, which is linked to heightened expression of genes associated with platelet aggregation and neutrophil-granulocyte degranulation (38). Prior research has demonstrated that certain clinical isolated strains of *Enterococcus faecium* possess the ability to induce human platelet aggregation (39) or activate human platelets *in vitro (* 40). Additionally, the ultrasonic extract derived from *Enterococcus faecium* has been found to impede cell cycle progression and suppress lymphocyte response (41). Hence, it was postulated that the dysregulation of *Enterococcus faecium* observed in elderly COVID-19 patients receiving PN may not be a mere bystander but rather a plausible contributor to the severity of COVID-19.

To summarize, our investigation revealed a heightened presence of *Enterococcus faecium*, a pathogenic agent responsible for COVID-19 in elderly individuals, in the context of PN administration. We conducted a more in-depth examination of the relationship between *Enterococcus faecium* and clinical prognosis. The findings revealed a positive correlation between *Enterococcus faecium* and the incidence of organ failure. Notably, a study demonstrated a robust association between gut bacteria and inflammation in COVID-19. Alterations in the gut microbiota were observed during the initial phases of infection, and there were reported correlations between the composition of the gut microbiota and levels of various proinflammatory cytokines known to be linked to COVID-19 (42). Furthermore, our study revealed a significant positive association between *Enterococcus faecium* and the quantities of white blood cells and neutrophils. This finding suggests that the administration of PN can influence alterations in the gut microbiota of elderly patients with COVID-19, resulting in the proliferation of the opportunistic pathogen *Enterococcus faecium* and subsequently impacting the clinical prognosis.

In the present study, it was observed that the abundance of multiple beneficial bacteria was notably higher in the group of patients receiving EN. Specifically, elderly COVID-19 patients with EN support exhibited a significant increase in the *Ruminococcus* and *Blautia* genera. It is worth noting that the genus *Blautia* has been shown to possess anti-inflammatory properties, as evidenced by previous studies (43, 44). Additionally, the genus *Ruminococcus* is recognized as a crucial contributor to the intestinal ecosystem, and probiotic strains derived from this genus may play a role in combating various pathogens within the same pathological niches (45). Multiple studies have consistently demonstrated that a decrease in the proportional prevalence of symbiotic bacteria is widely recognized as a crucial marker of ecological disequilibrium in inflammatory bowel disease, rheumatism, and multiple sclerosis (46, 47). Furthermore, a decline in *Ruminococcus* levels was observed among elderly COVID-19 patients receiving parenteral nutrition. Diminished quantities of *Ruminococcus* have been linked to various inflammatory bowel diseases and are pivotal in upholding intestinal well-being owing to their capacity to generate butyrate and other short-chain fatty acids (SCFA) (48). Among SCFA, butyrate exhibits anti-inflammatory properties, potentially elucidating the rationale behind the heightened severity of the disease in patients. Additional investigation into the association between *Ruminococcus* and clinical prognosis revealed a negative correlation between the relative abundance of *Ruminococcus* and complications experienced during hospitalization. The presence of gastrointestinal symptoms in COVID-19 patients has been linked to escalated disease severity and complications (49) with an excessive immune response to the virus believed to be a pivotal factor in driving disease progression (50). Our hypothesis posits that alterations in the microbial composition, particularly within the gastrointestinal tract, could potentially contribute to the pathogenesis of disease by actively participating in the emergence of complications. Maintaining a stable bacterial profile throughout hospitalization may exert a beneficial influence on the progression of the disease. Consequently, it is imperative to take into account the significance of a healthy and diverse gut microbiome in the therapeutic management of COVID-19. Furthermore, the disruption of metabolic functionality within the gut microbiota serves as a predictive indicator of disease severity among individuals afflicted with COVID-19.

Our study revealed variations in the relative prevalence of significant anticipated genomic functions within the gut microbiota between the enteral and parenteral nutrition cohorts. Notably, distinct metabolic markers within the same KEGG pathway exhibited disparities among samples obtained from patients receiving different forms of nutritional support, indicating a potential correlation with initial colonization and the advancement of disease. Consequently, modifications in the metabolic composition and energy generation of immune cells have the potential to impact their activation, thereby exacerbating the dysregulated immune response observed in individuals afflicted with COVID-19. In relation to functional biomarkers, our study revealed a significant activation of pathways associated with ribosomes, starch and sucrose metabolism, phosphotransferase system, ascorbate and uronate metabolism, as well as microbial metabolic pathways in various environments within the poor prognosis group, specifically referred to as the PN group. It is justifiable to deduce that the dysbiosis of microbiota caused by PN support in elderly patients with COVID-19 infection may substantially diminish crucial intestinal metabolic pathways and result in an escalation in the abundance of opportunistic microbiota. Consequently, the gut microbiota could potentially impact the prognosis of COVID-19 *via* metabolic pathway.

Our study encountered certain limitations. Firstly, the inclusion of patients treated at a solitary facility may restrict the generalizability of our findings to patients receiving treatment at other medical centers. Therefore, it is imperative to conduct multi-center studies encompassing large cohorts to examine alterations in intestinal microbiota among elderly patients infected with SARS-CoV-2 under varying nutritional support regimens. Secondly, the presence of heterogeneous patients and diverse clinical management approaches may introduce complexity in discerning microbial signatures associated with COVID-19. Hence, the impact of clinical management on the alteration of the gut microbiome composition in response to COVID-19 remains uncertain. Moreover, our analysis solely focused on the modifications in bacterial composition within the fecal samples of COVID-19 patients using 16S rRNA gene sequencing, without employing shotgun metagenomic sequencing. Consequently, further investigations are warranted to explore the variations in other microbiota constituents, such as viruses and fungi, in forthcoming research endeavors.

## Conclusions

In summary, our study shows that the changes in the composition of intestinal flora in elderly COVID-19 patients receiving different nutritional support strategies may be related to different clinical outcomes. The abundance of *Enterococcus_faecium* in elderly COVID-19 patients receiving PN is significantly increased, and is closely related to poor clinical outcomes. It highlights the potential of microbiome-centric interventions to mitigate and manage COVID-19 in older adults with different nutritional support options. Furthermore, we observed significant changes in gene function and metagenomic pathways in the microbiome, highlighting the potential role of the gut microbiome as a biomarker mediator.

## Data availability statement

The datasets presented in this study can be found in online repositories. The names of the repository/repositories and accession number(s) can be found in the article/supplementary material.

## Ethics statement

The studies involving humans were approved by the institutional ethics board of the Sixth Affiliated Hospital, Sun Yat-sen University. The studies were conducted in accordance with the local legislation and institutional requirements. The participants provided their written informed consent to participate in this study.

## Author contributions

JZ: Writing – original draft. JD: Writing – review & editing. JL: Writing – review & editing. YS: Writing – review & editing. JH: Writing – review & editing. DL: Writing – review & editing. MS: Writing – review & editing. YC: Writing – review & editing. SL: Writing – review & editing. XB: Writing – review & editing. ML: Writing – review & editing. TX: Writing – review & editing. QZ: Writing – review & editing. XG: Writing – review & editing.
